# Hydroxychloroquine‐Induced Bradycardia in a Male Patient With Sarcoidosis: A Case Report

**DOI:** 10.1002/ccr3.70986

**Published:** 2025-09-25

**Authors:** Sana Rasheed, Umaima Aijaz, Syeda Sakina Batool Naqvi, Ahmed Asad Raza, Abedin Samadi

**Affiliations:** ^1^ Department of Medicine Jinnah Sindh Medical University Karachi Pakistan; ^2^ Department of Medicine Kabul University of Medical Science Kabul Afghanistan

**Keywords:** bradycardia, cardiac conduction, case report, hydroxychloroquine, sarcoidosis

## Abstract

Hydroxychloroquine (HCQ) is often used in sarcoidosis as a steroid‐sparing agent. Though generally safe, rare cardiac side effects like bradycardia can occur, especially in patients with underlying cardiac vulnerability. A 40‐year‐old South Asian male with biopsy‐proven sarcoidosis, stable on HCQ, azathioprine, and prednisolone, presented with low‐grade fever, vomiting, and diarrhea. He was febrile, but hemodynamically stable. Labs showed leukocytosis, elevated CRP, and prerenal acute kidney injury. IV hydrocortisone was started, after which he developed sudden asymptomatic bradycardia (30–40 bpm). ECG revealed sinus bradycardia with partial right bundle branch block. Cardiac enzymes and echocardiogram were normal. Despite switching to methylprednisolone, bradycardia persisted. Given the temporal correlation and exclusion of other causes, HCQ‐induced bradycardia was suspected. HCQ was discontinued, and the heart rate normalized within 48 h. This case highlights the rare risk of HCQ‐induced bradycardia, which can unmask or worsen occult cardiac sarcoidosis, even after long‐term therapy. Clinicians should maintain vigilance for conduction abnormalities in sarcoidosis patients receiving HCQ, particularly when immunosuppressive regimens are modified.

**Summary**

- Hydroxychloroquine can rarely cause clinically significant bradycardia, especially in sarcoidosis patients with possible occult cardiac involvement.
- Early recognition and prompt drug withdrawal can rapidly reverse conduction abnormalities.

**Abbreviations**
AKI: acute kidney injury
AV node: atrioventricular node
BP: blood pressure
CRP: C‐reactive protein
DMARD: disease‐modifying antirheumatic drug
ECG: electrocardiogram
HCN: hyperpolarization‐activated cyclic nucleotide‐gated channels
HCQ: hydroxychloroquine
IV: intravenous
RA: rheumatoid arthritis
SA node: sinoatrial node
Th1: T‐helper 1
VHA: Veterans Health Administration

## 1 Introduction

Sarcoidosis is a granulomatous inflammatory disease that involves multiple organ systems of the human body. Though the etiological mechanisms are still obscure, both genetic and environmental factors, particularly infections and immunological reactions, are believed to contribute to the development of this disease [1]. The inflammation in sarcoidosis results from an overloaded reaction of Helper T1 (Th1) cells to an undiscovered antigen, leading to the recruitment of many Th1 cells and macrophages that develop into epithelioid cells and form self‐limiting noncaseating granulomas, especially in lymphoid tissues [2, 3]. A study on sarcoidosis in US veterans, based on the data from the Veterans Health Administration (VHA) from 2009 to 2013, recognized black race, female gender, and tobacco usage as factors increasing the risk of this disease [4]. While some patients remain asymptomatic, in others, it may manifest in the form of cardiac, cutaneous, pulmonary, ocular, or neuro‐sarcoidosis. Hydroxychloroquine (HCQ) is now considered a potential treatment option for sarcoidosis, especially in cases where glucocorticoids cannot be used because of their adverse effects. HCQ administration for treating cutaneous sarcoidosis showed a 55% efficacy rate after 24 weeks of treatment, but 41.7% of individuals reported experiencing side effects that led to HCQ discontinuation in 21.7% of cases [5]. A study revealed that HCQ influences pacemaker current, L‐type Ca + 2 current, and transient outward potassium current, along with fluctuations in intracellular Ca + 2 management disturbing the normal pacemaker activity and causing bradycardia [6].

Here, we report the case of a 40 year old male, a known case of sarcoidosis who suffered from HCQ‐induced bradycardia. This case emphasizes the importance of closely monitoring patients for rare but potentially serious side effects of medications. In this instance, the prompt cessation of HCQ led to stabilized reversion of the patient's bradycardia and prevented any irreversible heart damage.

## 2 Case History

A 40‐year‐old man with known sarcoidosis diagnosed several years earlier on the basis of biopsy and clinical presentation presented with a history of low‐grade fever, vomiting, and diarrhea for three days. He did not have chest pain, shortness of breath, joint pain, or skin lesions at the time of admission. His clinical status remained stable over the last few years on oral HCQ 250 mg twice a day, azathioprine 50 mg once a day, and prednisolone 10 mg once a day. On Day 1 of admission, his physical workup revealed a febrile patient (38.2°C) with mild dehydration. His pulse was 110 bpm and blood pressure was 128/86 mmHg. There was no respiratory difficulty. Systemic examination, including cardiovascular and abdominal systems, was normal.

Early blood work was notable for leukocytosis, raised CRP, mild hypoalbuminemia, prerenal AKI, and trace proteinuria (Table 1). A presumptive diagnosis of infective gastroenteritis with AKI secondary to dehydration was made. Intravenous fluids and IV ceftriaxone (2 g daily) were used in the management.

**TABLE 1:** Laboratory parameters on admission showing inflammatory markers and renal involvement.

Parameter	Result	Reference range
White blood cell count	14.8 × 10^9^/L	4.0–11.0 × 10^9^/L
Hemoglobin	12.6 g/dL	13.5–17.5 g/dL (male)
Platelet count	240 × 10^9^/L	150–450 × 10^9^/L
C‐reactive protein (CRP)	68 mg/L	< 5 mg/L
Serum creatinine	1.8 mg/dL	0.7–1.3 mg/dL
Blood urea nitrogen (BUN)	32 mg/dL	7–20 mg/dL
Serum sodium	134 mmol/L	135–145 mmol/L
Serum potassium	3.9 mmol/L	3.5–5.0 mmol/L
Serum albumin	3.0 g/dL	3.5–5.5 g/dL
Urine protein (dipstick)	+1	Negative
ALT (SGPT)	32 U/L	< 45 U/L
AST (SGOT)	28 U/L	< 40 U/L

Given his proteinuria and underlying sarcoidosis, an inciting flare was also a consideration. Later on Day 1, IV hydrocortisone 200 mg tid was started as stress‐dose steroids, chosen over oral prednisolone because the patient was acutely unwell with vomiting, where oral absorption would be unreliable, and a rapid systemic effect was desired. Azathioprine and HCQ were maintained. Oral prednisolone was held briefly.

**FIGURE 1:**
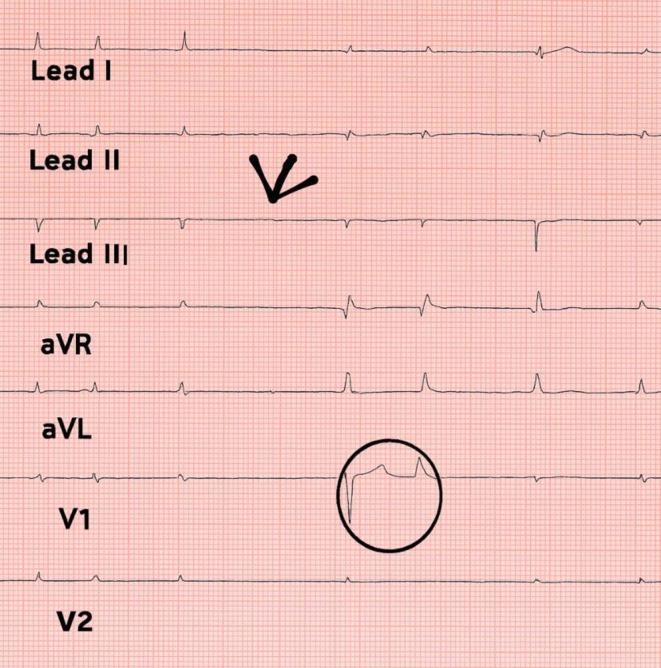
ECG of the patient showing sinus bradycardia (arrow) and right bundle branch block (circle).

Within minutes of the first dose of hydrocortisone administration, the patient presented with significant bradycardia, with his heart rate reducing to 30–40 bpm. He was asymptomatic and hemodynamically stable (BP 140/70 mmHg) despite the low heart rate. The ECG showed sinus bradycardia with partial right bundle branch block and no ischemic changes (Figure 1). Cardiac enzymes were within the normal range.

**FIGURE 2:**
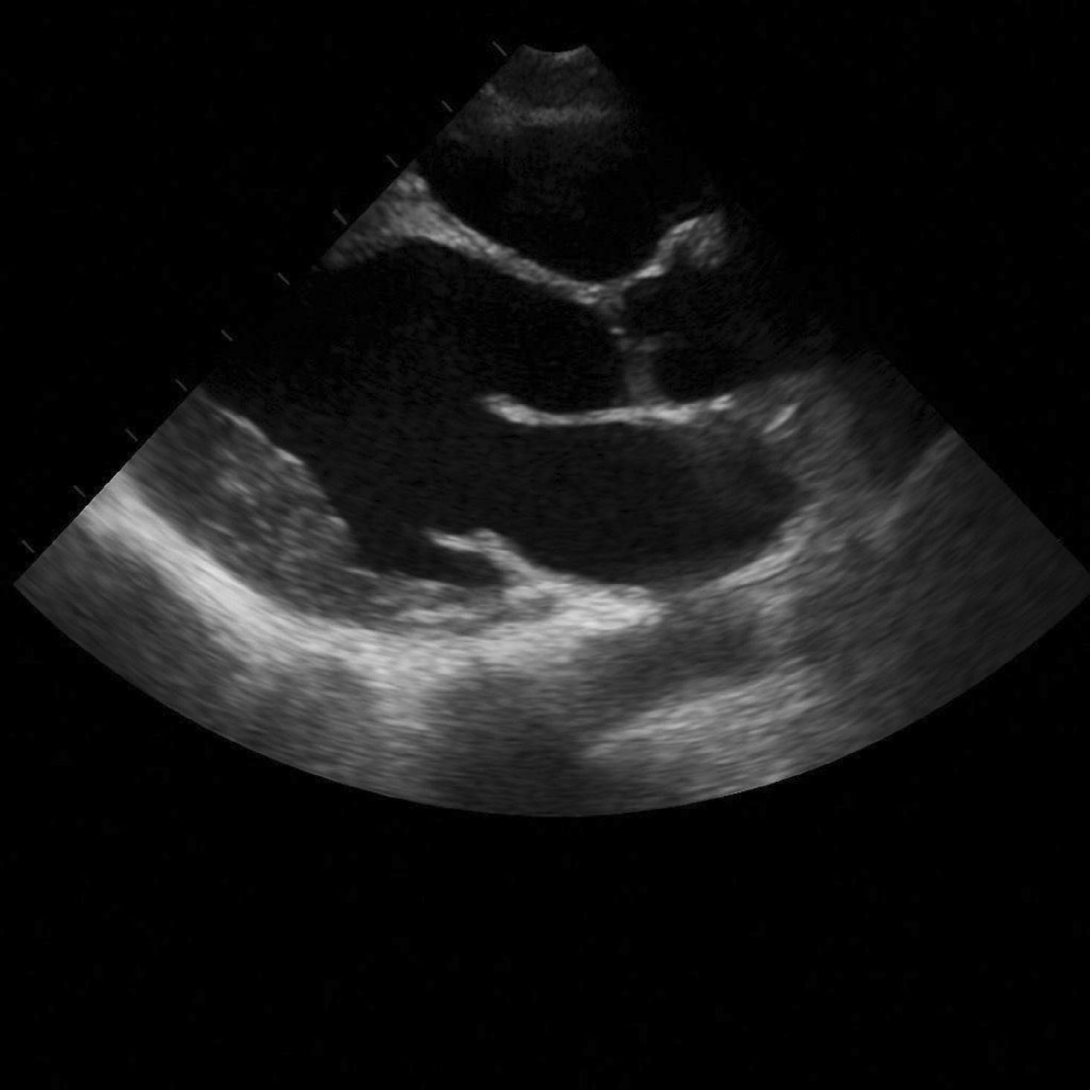
Echocardiogram of the patient showing normal left ventricular function without structural abnormalities, helping to differentiate hydroxychloroquine‐induced bradycardia from structural cardiac involvement of sarcoidosis.

On Day 1–2, hydrocortisone was halted and substituted with IV methylprednisolone 50 mg daily, but the bradycardia was continuing. Because of the temporal relation and absence of other causative elements, HCQ‐induced bradycardia was highly suspected, and HCQ was discontinued permanently. Within the following 48 h, the heart rate of the patient progressively returned to baseline. There were no recurring episodes. The bedside echocardiogram revealed normal left ventricular performance with no structural disease (Figure 2).

The patient was stable and discharged on Day 3. His prednisolone oral was up‐titrated to 30 mg daily (taper) and azathioprine 50 mg daily was maintained as a steroid‐sparing drug. Hydroxychloroquine was discontinued forever and methotrexate or leflunomide were looked into as the likely substitutes, in case the disease activity in the follow‐up visit was found to rise. He was also administered oral cefuroxime 250 mg twice daily to cover for the rest of the antibiotics. A chronological overview of the patient's clinical course, intervention, and resolution of symptoms is summarized in Table 2.

**TABLE 2:** Chronological overview of clinical course, interventions, and outcomes.

Hospital day	Clinical events	Interventions	Outcomes
Day 1 (admission)	Presented with 3 days of fever, vomiting, diarrhea. Stable vitals; mild dehydration. Labs: leukocytosis, ↑CRP, prerenal AKI, trace proteinuria	IV fluids; IV ceftriaxone 2 g daily. Oral prednisolone held. Continued azathioprine & HCQ	Diagnosed with infective gastroenteritis + dehydration‐related AKI; sarcoidosis flare considered
Day 1 (later)	Started on IV hydrocortisone 200 mg tid for suspected flare	Hydrocortisone initiated	Within minutes developed asymptomatic bradycardia (30–40 bpm); ECG: sinus bradycardia + partial RBBB
Day 1–2	Bradycardia persisted despite switching steroids	Hydrocortisone stopped; IV methylprednisolone 50 mg daily started	No improvement in bradycardia
Day 2	HCQ‐induced bradycardia suspected	HCQ permanently discontinued	Heart rate progressively improved
Day 3	HR normalized; echocardiogram normal	Oral prednisolone restarted at 30 mg daily (taper planned). Azathioprine continued. Antibiotics switched to oral cefuroxime 250 mg bid	Clinically stable, no recurrence of bradycardia. Discharged

## 3 Differential Diagnosis

Several differential diagnoses were considered in this patient with sarcoidosis who presented with fever, gastrointestinal upset, acute kidney injury, and bradycardia, and these are summarized in Table 3.

**TABLE 3:** Comparative differential diagnosis in a patient with sarcoidosis and bradycardia.

Differential diagnosis	Supporting features	Arguing against/reason for exclusion
Infective gastroenteritis with prerenal AKI	Vomiting, diarrhea, dehydration, leukocytosis, elevated CRP	Resolution after drug withdrawal; no ongoing infection identified
Sarcoidosis flare (renal or cardiac involvement)	Sarcoidosis can cause granulomatous interstitial nephritis or conduction abnormalities	No systemic activity; symptoms resolved after hydroxychloroquine withdrawal
Steroid‐induced bradycardia	Temporal association with intravenous hydrocortisone	Bradycardia persisted after switch to methylprednisolone
Hydroxychloroquine‐induced bradycardia	Known risk of sinus node dysfunction; resolution after discontinuation	Strong temporal relationship; no other cause identified
Azathioprine or infection‐related cardiotoxicity	On immunosuppressive therapy; systemic illness	Normal cardiac enzymes and echocardiography
Electrolyte disturbances (secondary to diarrhea)	GI losses could contribute	No electrolyte derangements documented

## 4 Patient Perspective

I was admitted with fever and stomach issues and was treated with antibiotics and steroids. During my stay, my doctors identified a heart rate problem related to one of my long‐term medicines, which they stopped. I am satisfied with the care I received and thankful that my condition was managed safely and explained clearly to me.

## 5 Discussion

We present a unique case of sarcoidosis in a male patient who suffered from bradycardia due to the use of HCQ. He presented with low‐grade fever, vomiting, and diarrhea. He was on HCQ, azathioprine, and prednisolone over the last few years. Recently, instead of IV prednisolone, cortisone was started due to the problem of proteinuria, and since then, significant bradycardia was noted. ECG showed sinus bradycardia with partial right bundle branch block. By the help of drug discontinuation, a causal association was made between HCQ and bradycardia. Although HCQ is considered a safe immunosuppressant, this case highlights its rare cardiovascular side effect.

The fact that HCQ can cause bradycardia is also supported by other studies. For example, Nguyen et al. conducted an observational, retrospective study using VigiBase and reported that atrioventricular and bundle branch blockages, cardiac arrest, and conduction abnormalities have all been linked to HCQ. Its prolonged exposure over a number of months was also linked to possibly fatal heart failure [7]. Capel et al. [8] reported that through channels that block the “funny” current (If), HCQ reduces the rate at which action potentials fire in sinoatrial nodes. Similarly, Thaper et al. [9] described a HCQ overdose case that, despite intensive care, showed a rapidly escalating intraventricular conduction delay and QT prolongation, leading to severe bradycardia and shock. A case of an 84‐year‐old woman with COVID was presented by Kang et al. (2020). She experienced sinus bradycardia and QTc prolongation when HCQ was included in her medications, and symptoms were reversed when it was discontinued [10]. Keating et al. (2005) presented a case of a 39‐year‐old woman with systemic lupus erythematosus, who experienced conduction anomalies and chest pain after the use of HCQ. Ventricular thickness and systolic dysfunction were discovered during an echocardiogram [11]. Similarly, Teixeira et al. (2002) described the example of a 58‐year‐old rheumatoid arthritis (RA) patient, whose long‐term usage of chloroquine caused significant and irreparable heart damage. Her total atrioventricular block, which was confirmed by an electrophysiological study, was the cause of her syncopal episodes. The study determined that the cardiac changes were irreversible and that a permanent pacemaker was necessary [12].

Conversely, other studies have highlighted the safety of HCQ in patients without structural heart disease. A retrospective cohort study on RA patients was conducted by Faselis et al. (2021), and results show that during the first year following the start of HCQ or another nonbiologic DMARD, there is a low prevalence of long QT syndrome and hospitalization for arrhythmias. There is no proof that HCQ treatment raises the risk of fatalities or other unfavorable cardiovascular events [13]. A cross‐sectional study was conducted by Carmichael et al. [14], and no relation was found between HCQ concentration, dose, and ECG changes or bradycardia.

We suggest that HCQ‐induced bradycardia can be a result of two mechanisms:

1. The specific pharmacodynamic influence of HCQ on cardiovascular pacemaker activity.
2. The existence of systemic cardiac involvement brought on by sarcoidosis, which may cause the heart to be more susceptible to such effects.

As discussed previously, HCQ blocks the HCN channels that are responsible for the regulation of sinoatrial node funny current, which ultimately led to the slowing of heart rate. This effect is mild and not noticed in otherwise healthy people, but the patients with underlying inflammatory myocardial diseases or conduction disorders go through a greater response. Moreover, it is well known that HCQ and its analogue chloroquine build up in lysosomes, disrupt ion homeostasis, and inhibit hERG channels to stop the inward rectifier potassium current (IKr). Bradyarrhythmia, conduction delay, and QTc lengthening result from this prolonged action potential duration [8]. These direct electrophysiological effects have been linked to ventricular arrhythmias, sinus bradycardia, and AV block in case reports of patients with lupus, rheumatoid arthritis, and COVID‐19. On the other hand, sarcoidosis can cause fibrosis of the conduction system of the heart, specifically His‐Purkinje system, AV node, and SA node. HCQ may bring latent conduction problems to clinical notice or lower the threshold for arrhythmia in patients with systemic sarcoidosis. As an example, baseline ECG and ventricular abnormalities are found in around 20%–30% of patients with asymptomatic extracardiac sarcoidosis [15]. Given that, there is a possibility that our patient had cardiac sarcoidosis and it was clinically silent until it was revealed by the effect of HCQ. The reversibility of bradycardia symptoms after the discontinuation of HCQ further supports this fact. Even though bradyarrhythmia and HCQ use happened at the same time, causality is still presumed because, without cardiac MRI, PET, or electrophysiologic measurements, we are unable to conclusively link the arrhythmia to HCQ instead of sarcoid‐related heart disease.

Given that HCQ may have an impact on cardiac conduction, physicians should exercise caution when treating patients on long‐term medication, especially if they have systemic sarcoidosis or other arrhythmia risk factors. A baseline electrocardiogram and periodic rhythm monitoring are recommended [15]. Patients should be instructed to report palpitations, dizziness, or syncope as soon as possible [16]. To sum up, this case emphasizes the necessity of increased clinical monitoring. Rarely, even commonly prescribed drugs like HCQ can cause potentially fatal conduction abnormalities, which serves as a reminder to doctors to weigh the potential therapeutic benefits against close observation, particularly in patients who are already at the risk for heart disease.

## 6 Conclusion

This case investigation highlights the significance of safety measures for the preservation of myocardial function as one of the primary factors that should be taken into account while prescribing HCQ for the treatment of sarcoidosis. In cases where patients are considering steroid‐sparing therapy, clinicians must evaluate the advantages and possible hazards of HCQ to minimize the risk of associated cardiac complications, especially in patients with a preexisting history of myocardial damage and cardiovascular diseases. Moreover, thorough surveillance, providing personalized treatment regimens, and additional research are crucial to gain a better understanding of this disease, the underlying mechanisms of HCQ‐associated bradycardia, and ways to prevent such undesirable effects for the successful management of sarcoidosis.

## Author Contributions

**Sana Rasheed:** project administration, supervision, validation, visualization, writing – original draft, writing – review and editing. **Umaima Aijaz:** conceptualization, visualization, writing – original draft, writing – review and editing. **Syeda Sakina Batool Naqvi:** project administration, writing – original draft, writing – review and editing. **Ahmed Asad Raza:** project administration, writing – original draft, writing – review and editing. **Abedin Samadi:** project administration, writing – original draft, writing – review and editing.

## Ethics Statement

Our institution does not require ethical approval for reporting individual case reports.

## Consent

The patient gave a verbal informed consent to participate in the study and use their anonymized information. Written informed consent was obtained from the patient for publication of this case report and any accompanying images. A copy of the written consent is available for review by the Editor‐in‐Chief of this journal.

## Conflicts of Interest

The authors declare no conflicts of interest.

## Data Availability

Data supporting the findings of this case report are available from the corresponding author upon reasonable request, subject to institutional and ethical guidelines.

## References

[1] P. Brito‐Zerón , R. Pérez‐Álvarez , and M. Ramos‐Casals , “Sarcoidosis,” Medicina Clínica 159, no. 4 (2022): 195–204, 10.1016/j.medcli.2022.03.009. 35680449

[2] V. D. Serei and B. Fyfe , “The Many Faces of Cardiac Sarcoidosis,” American Journal of Clinical Pathology 153, no. 3 (2019): 294–302, 10.1093/ajcp/aqz169. 31769474

[3] “The Roles of T Helper 1, T Helper 17 and Regulatory T Cells in the Pathogenesis of Sarcoidosis,” (2016), https://pubmed.ncbi.nlm.nih.gov/27921415/.

[4] M. I. Seedahmed , A. D. Baugh , M. T. Albirair , et al., “Epidemiology of Sarcoidosis in U.S. Veterans From 2003 to 2019,” Annals of the American Thoracic Society 1, no. 6 (2023): 797–806, 10.1513/annalsats.202206-515oc. PMC10257030 36724377

[5] B. Vermeer , M. Veltkamp , and A. D. Vorselaars , “Effect of Hydroxychloroquine in Cutaneous Sarcoidosis or Musculoskeletal‐Related Pain,” In: 1203 – Sarcoidosis and Other Granulomatous ILD/DPLD, (European Respiratory Society, 2022), cited Sep 5, 2025, p. 763, https://publications.ersnet.org/lookup/doi/10.1183/13993003.congress‐2022.763.

[6] S. Segal , L. Arbel‐Ganon , S. Mazgaoker , M. Davoodi , and Y. Yaniv , “Increase in CA2+‐Activated CAMP/PKA Signaling Prevents Hydroxychloroquine‐Induced Bradycardia of the Cardiac Pacemaker,” Frontiers in Physiology 13 (2022): 839140, 10.3389/fphys.2022.839140. 35634151 PMC9130770

[7] L. S. Nguyen , C. Dolladille , M. D. Drici , et al., “Cardiovascular Toxicities Associated With Hydroxychloroquine and Azithromycin,” Circulation 142, no. 3 (2020): 303–305, 10.1161/circulationaha.120.048238. 32442023 PMC7365677

[8] R. A. Capel , N. Herring , M. Kalla , et al., “Hydroxychloroquine Reduces Heart Rate by Modulating the Hyperpolarization‐Activated Current if: Novel Electrophysiological Insights and Therapeutic Potential,” Heart Rhythm 12, no. 10 (2015): 2186–2194, 10.1016/j.hrthm.2015.05.027. 26025323 PMC4689153

[9] A. Thaper , S. J. Ross , Z. Agarwal , M. Langston , W. M. Miles , and A. Austin , “Progression of ECG in Hydroxychloroquine Overdose,” Journal of Electrocardiology 77 (2023): 68–71, 10.1016/j.jelectrocard.2023.01.002. 36652870

[10] Y. Kang , H. Wang , H. Chen , et al., “Suspected Hydroxychloroquine‐Induced Sinus Bradycardia and QTc Prolongation in a Patient With COVID‐19,” International Heart Journal 61, no. 5 (2020): 1056–1058, 10.1536/ihj.20-271. 32921678

[11] R. J. Keating , S. Bhatia , S. Amin , A. Williams , L. J. Sinak , and W. D. Edwards , “Hydroxychloroquine‐Induced Cardiotoxicity in a 39‐Year‐Old Woman With Systemic Lupus Erythematosus and Systolic Dysfunction,” Journal of the American Society of Echocardiography 18, no. 9 (2005): 981.e1–981.e5, 10.1016/j.echo.2005.01.012. 16153529

[12] R. A. Teixeira , M. M. Filho , L. A. Benvenuti , R. Costa , A. A. Pedrosa , and S. a. D. Nishióka , “Cardiac Damage From Chronic Use of Chloroquine: A Case Report and Review of the Literature,” Arquivos Brasileiros de Cardiologia 79, no. 1 (2002): 85–88, 10.1590/s0066-782x2002001000009. 12163948

[13] C. Faselis , Q. Zeng‐Treitler , Y. Cheng , et al., “Cardiovascular Safety of Hydroxychloroquine in US Veterans With Rheumatoid Arthritis,” Arthritis and Rheumatology 73, no. 9 (2021): 1589–1600, 10.1002/art.41803. 33973403

[14] S. J. Carmichael , R. O. Day , and S. E. Tett , “A Cross‐Sectional Study of Hydroxychloroquine Concentrations and Effects in People With Systemic Lupus Erythematosus,” Internal Medicine Journal 43, no. 5 (2013): 547–553, 10.1111/imj.12100. 23425382

[15] D. H. Birnie , P. B. Nery , A. C. Ha , and R. S. B. Beanlands , “Cardiac Sarcoidosis,” Journal of the American College of Cardiology 1, no. 4 (2016): 411–421, 10.1016/j.jacc.2016.03.605. 27443438

[16] D. M. Roden , R. A. Harrington , A. Poppas , and A. M. Russo , “Considerations for Drug Interactions on QTC in Exploratory COVID‐19 Treatment,” Circulation 141, no. 24 (2020): e906–e907, 10.1161/circulationaha.120.047521. 32267732

